# Zinc induces caspase-dependent mitochondrial pathway of the programmed cell death in haemocytes of *Drosophila melanogaster*

**DOI:** 10.1007/s10534-012-9530-1

**Published:** 2012-02-25

**Authors:** Marta Filipiak, Grzegorz Tylko, Elzbieta Pyza

**Affiliations:** Department of Cell Biology and Imaging, Institute of Zoology, Jagiellonian University, Gronostajowa 9, 30-387 Kraków, Poland

**Keywords:** Apoptosis, Metallothioneins, Mitochondria, Haemolymph, Fruit fly

## Abstract

Zinc is an essential trace element in cells. However, its high level in cytoplasm promotes activation of stress signaling pathways and may lead to cell death. In the present study we used *Drosophila melanogaster* blood cells (haemocytes), obtained from the third instar larvae, to study the effects of high concentrations of Zn^2+^ on programmed cell death (PCD). We analyzed the activity of caspases, the level of caspase inhibitor protein DIAP1 and metallothioneins, as well as calcium concentrations and activity of mitochondria in haemocytes exposed to 0.35 and 1.7 mM concentrations of Zn. The obtained results showed that rapid increase of [Zn^2+^]_*i*_ in the cytoplasm up-regulates metallothionein *MtnB* but not *MtnA* gene expression in cells treated with Zn^2+^ in both concentrations. Excess of Zn^2+^ also induced activation of the initiator caspase Dronc, associated with the mitochondrial pathway of PCD, and the effector caspase DrICE. In turn, the activity of receptor-regulated Dredd caspase was not changed. The level of DIAP1 decreased significantly in haemocytes in the presence of high Zn^2+^ concentration in comparison to untreated cells. Moreover, mitochondrial membrane potential was significantly decreased after exposure to Zn ions. These results indicate that high concentration of Zn^2+^ in the cytoplasm of haemocytes induces PCD via a mitochondrial pathway and that caspases play a pivotal role in this process.

## Introduction

Divalent zinc cations (Zn^2+^) are present in all living organisms and are important in development and metabolism of cells. Zn^2+^ is a cofactor of more than 300 enzymes and a structural component of thousands of transcription factors (John et al. 2010). Zinc acts as a second messenger or a neurotransmitter, participating in gene expression, DNA/protein synthesis and in extracellular signal transduction. Thus, it is involved in cell growth, differentiation, proliferation and especially, in cell survival or death (Wellinghausen et al. 1997; Rink and Gabriel 2000; Bayersmann and Haase 2001).

The physiological concentration of Zn ions is controlled by endogenous mechanisms and differs depending on cell type. The mechanisms that regulate the intracellular Zn^2+^ level are very complex and generally based on zinc interactions with specific cytoplasmic proteins, e.g., metallothioneins (MTs). In result, the free cytoplasmic fraction of [Zn^2+^]_*i*_ is maintained at a low level (Tubek 2007; Lichten and Cousins 2009). Although Zn ions are usually thought as being relatively harmless, any fluctuations of [Zn^2+^]_*i*_ affect cell activity and promote cell death (Truong-Tran et al. 2000). Especially, cells of the immune system are sensitive to changes in Zn ion concentration. Deficiency of Zn^2+^ results in increased susceptibility of immune cells to pathogens and in a consequence, leads to immune disorders like lymphopenia or thymus atrophy (Fraker et al. 2000). Even a mild decrease in Zn^2+^ concentration depresses the activity of immune cells. For example, cytotoxicity of NK cells is significantly impaired, phagocytotic activity of macrophages and neutrophils is reduced, B cells show apoptotic features whereas T cells are stimulated to auto- and alloreactivity (Ibs and Rink 2003).

In cell survival or death decisions, Zn^2+^ plays a dual role. This role depends on both cell-specificity and metal concentration in the extracellular space and in the cytoplasm. It has been reported that low concentrations of Zn ions in the cell environment suppress apoptosis in many cell types stimulated to enter programmed cell death (PCD) pathways (Flieger et al. 1989; Fraker and Telford 1997; Perry et al. 1997; Aiuchi et al. 1998; Neves et al. 1998; Ze-peng et al. 2005). On the other hand, high concentrations of Zn^2+^ might induce PCD, the effects normally observed in Zn-deficiency. Proapoptotic activity of Zn^2+^ has been observed in different cell types either as a consequence of exogenous administration of Zn ions or after their release from cytoplasmic stores (Zhang et al. 2004; Knoch et al. 2008). It has also been reported that Zn^2+^ highly interacts with DNA, decreases mitochondrial membrane potential, impairs the stability of lysosomes, and inhibits proteins from IAP family. These events are sufficient to induce PCD (Sensi et al. 2003; Lee et al. 2009; Ku et al. 2010; Rudolf and Červinka 2010).

Haemocytes of *Drosophila melanogaster* take part in immune responses. They circulate in the haemolymph and their main role is the elimination of pathogens and parasites. In *D. melanogaster,* haemocytes are divided into three types: plasmatocytes, crystal cells and lamellocytes. The most numerous cells are plasmatocytes that functionally resemble mammalian macrophages. These cells participate in phagocytosis and production of antimicrobial peptides. Crystal cells possess crystalline cytoplasmic inclusions that contain phenoloxidase, necessary for melanization and wound repair. The lamellocytes are only present in larvae and appear after infection of larvae with parasites (Hoffman 1995; Echalier 1997; Lavine and Strand 2002; Meister 2004). Generally, the studies on *D. melanogaster* haemolymph cells, obtained from healthy larvae, are based on plasmatocytes. Lamellocytes are very rare whereas crystal cells degenerate within a few minutes after haemolymph collection (Ribeiro and Brehélin 2006).

Programmed cell death pathways in *D. melanogaster* and mammalian cells have the same core components (Abrams 1999). The major cysteine protease in *D. melanogaster* is the initiator caspase Dronc (homologue of the mammalian caspase-9) that is autoactivated in a protein complex called apoptosome (Dorstyn et al. 1999). Another caspase, DrICE, the mammalian caspase-3 homologue, is responsible for massive substrate cleavage and cell degradation (Song et al. 2000). In turn, Dredd is a homologue of mammalian initiator caspase-8 and it probably takes part in receptor-mediated PCD. However, it has been suggested that immune responses of cells require Dredd activity (Siegel 2006). The regulation of *D. melanogaster* caspase activity is under control of IAP proteins: DIAP1, DIAP2 and Deterin but only DIAP1 inhibits all death enzymes (Richardson and Kumar 2002).

As we have already reported (Szczerbina et al. 2008; Filipiak et al. 2010; Borowska and Pyza 2011), haemocytes isolated from wandering larvae of the third larval instar represent an excellent model to study the impact of many substances on cell-dependent immune responses. We have found that PCD of haemocytes is enhanced in the presence of high concentrations: 0.35 and 1.7 mM, of Zn ions. In our earlier study, we have observed that proapoptotic activity of Zn^2+^, revealed by phosphatidylserine exposure in the external leaflet of the cell membrane, is independent on caspase activity but we have studied total activity of either initiator and effector caspases using pan-caspase detection system.

The aim of the present study was to investigate Zn^2+^ content in haemocyte cytoplasm after exposure of cells to 0.35 and 1.7 mM Zn^2+^ and effects of both concentrations on: metallothionein gene expression (*MtnA* and *MtnB*), activity of caspases Dredd, Dronc and DrICE, the level of caspase inhibitor DIAP1, mitochondrial membrane potential, and on Ca^2+^ concentration in haemocytes.

## Materials and methods

### Animals

Adult fruit flies (*D. melanogaster*), Canton S, were obtained from the laboratory stock, kept in plastic flasks of 0.175 dm^3^ volume and fed with a standard cornmeal medium. Fruit flies were maintained at a constant temperature (24 ± 1°C) and in a light:dark cycle (LD 12:12). Females laid eggs in the feeding medium for 24 h and then adults were removed. Larvae were reared until the wandering stage in the third larval instar.

### Haemocytes isolation and Zn exposure

The third instar larvae of *D. melanogaster* were thoroughly washed in Hank’s Balanced Salt Solution (HBSS), pH 7.2, decapitated individually with a springbow dissecting microscissors and 20 μl of haemolymph was collected with a glass capillary (Microcaps, Drummand Scientific, USA). To maintain the number of haemocytes at 4 × 10^6^ per 1 ml, 20 μl of haemolymph was suspended in 280 μl of calcium free HBSS, pH 7.2. The number of haemocytes in diluted samples was established on the basis of several measurements by means of flow cytometry. The cell suspension was finally divided into four groups: (1) exposed to 0.35 mM of Zn^2+^, (2) exposed to 1.7 mM of Zn^2+^, (3) control without Zn^2+^ exposure, (4) positive or negative controls depending on the experiment. Before the treatments, cells were isolated from larval haemolymph by centrifugation at 1,500×*g* for 15 min. Zinc ion solutions used in the study were prepared from ZnCl_2_ (Merck, Germany). Finally, haemocytes were incubated for 3 h at 22°C in darkness, while slowly shaken in Rotomix 50800 (Thermolyne, USA).

### Measurements of intracellular Zn^2+^ concentration

Evaluation of [Zn^2+^]_*i*_ in haemocytes was performed using a fluorescent indicator FluoZin™-3, AM ester (Molecular Probes, USA) and analyzing images obtained with a confocal microscope (Zeiss LSM 510 META, Zeiss, Germany). Haemocytes suspended in the Schneider’s medium were placed onto glass microscope coverslips and allowed to adhere to the glass surface for about 1 h. Then, cells were treated with two concentrations of Zn ions in HBSS for 3 h. After that, an excess of Zn^2+^ was removed by washing the cells with HBSS and haemocytes were loaded with 5 μM solution of FluoZin-3 in HBSS, and incubated in darkness for 30 min. After loading, haemocytes were washed with HBSS and fluorescent images were taken with the confocal microscope. Fluorescence intensity of individual cells was measured using LSM-FCS software (Zeiss, Germany) and [Zn^2+^]_*i*_ was calculated from the mean fluorescence intensity of cells according to the formula: [Zn^2+^]_*i*_ = K_d_ × [(F − F_min_)/(F_max_ − F)], where FluoZin-3 K_d_(Zn^2+^) = 15 nM. Maximal (F_max_) and minimal (F_min_) values of FluoZin-3 fluorescence were estimated for the cells treated with 100 μM solution of zinc ionophore–pyrithione (2-mercaptopyridine-1-oxide sodium salt, Sigma–Aldrich, Germany) in HBSS in the presence of 2 mM Zn^2+^ and 100 μM solution of zinc chelator TPEN (tetrakis-(2-pyridylmethyl)-ethylenediamine, Molecular Probes, USA), respectively (Haase et al. 2006).

### Real time PCR analysis of *MtnA* and *MtnB* gene expressions

The concentration of RNA in Zn-treated and control group of haemocytes was assessed using a spectrophotometer (NanoDropMolecular Probes, USA™2000, ThermoSCIENTIFIC, USA) after treatment of cells with Trizol Reagent (Invitrogen, USA), according to the manufacturer’s protocol. Total RNA (1 μg) was converted into cDNA by means of reverse transcription system containing oligo(dT) and SuperScriptIII First-Strand Synthesis System for RT-PCR (Invitrogen, USA) in 20 μl reaction volume. The Real Time PCR amplification was performed with a Step One Plus System (Applied Biosystems, USA) using the mixture of diluted cDNA template (5 μl, 1:3 solution of nuclease free water), Power SYBR Green Master Mix (Applied Biosystems, USA), and gene-specific primers. The primers: *MtnA, MtnB* and *rpl32* (Ribosomal Protein L32) were designed using Primer-BLAST software (NCBI, USA) and synthesized at GenoMed (Poland). The following *D. melanogaster* primer sequences were utilized: metallothionein A, forward, 5′-TGCGGAAGCGGTAAGTTCGCAG-3′, reverse, 5′-ATTTCTTGTCGCCGCCGCACT-3′ (product length: 352 bp), metallothionein B, forward, 5′-GCCTCAGCCAAGTGAAAGTCGAGA-3′, reverse, 5′-ACAGCTTGCTGCTGCGTTGT-3′ (product length: 94 bp), and Ribosomal Protein L32, forward, 5′-AGAAGCGCAAGGAGATTGTC-3′, reverse, 5′-ATGGTGCTGCTATCCCAATC-3′ (product length: 233 bp). Changes in metallothionein gene expressions in Zn-treated haemocytes in relation to the control group were based on the ∆∆*C*_T_ method (Livak and Schmittgen 2001) in the presence of normalizing *rpl32* gene.

### Estimation of caspase Dredd, Dronc and DrICE activity

The activities of selected *D. melanogaster* caspases were measured in haemocytes using Caspase-Glo 3/7 Assay for caspase DrICE, Caspase-Glo 9 Assay for caspase Dronc and Caspase-Glo 8 Assay (Promega, USA) for caspase Dredd. As the positive control in this experiment, the fruit fly’s haemocytes were treated with 1 μM staurosporine (Sigma-Aldrich, Germany) for 3 h. Next, the control group of cells and groups treated with Zn were lysed for 30 min at 4°C with a buffer containing Tris–HCl, pH 7.5 (20 mM, Merck, Germany), sodium chloride (150 mM, Chempur, Poland), Na_2_EDTA (1 mM, POCH, Poland), EGTA (1 mM, Sigma, Germany), 1% Triton X-100 (Calbiochem, USA), sodium pyrophosphate (2.5 mM), β-glycerophosphate (1 mM). Then, the samples were centrifuged at 1,500×*g* for 15 min at 4°C, supernatant was collected and protein content was estimated using Micro BCA Protein Kit (Thermo Scientific, USA). The solutions with 30 μg of protein were prepared and mixed with appropriate caspase reagent in 1:1 ratio, according to the manufacturer’s protocol. After 1 h of incubation at room temperature in darkness, luminescence of the samples was measured using a luminometer (GloMax^®^ 20/20 Single Tube Luminometer, Promega, USA).

### Determination of DIAP1 protein level by immunocytochemistry

100 μl of haemocyte suspensions from both control groups and Zn-treated ones were dropped onto glass coverslips and left to adhere for 1 h. Then all experimental groups were fixed with 2% formaldehyde solution in phosphate buffer (PBS) (pH 7.4) for 10 min at room temperature. Fixed specimens were thoroughly washed with PBS and additionally permeabilized with 0.1% Triton X-100 in PBS for 5 min. Next, 3% BSA in PBS was used to block unspecific antibody binding for 30 min and immunolabeled overnight at 4°C with the mouse primary monoclonal antibodies against DIAP1 (Abcam, USA), diluted 1:1,000 in 3% BSA solution in PBS. The excess of antibodies was removed by washing with PBS and then the haemocytes were incubated with 3% BSA in PBS for 30 min. The secondary goat anti-mouse antibodies Cy™3-conjugated (Jackson ImmunoResearch, USA) were finally used to label DIAP1 protein. The specimens were rinsed with PBS before mounting with Vectashield Mounting Medium for Fluorescence (Vector Laboratories Inc., USA). Confocal images were taken and the fluorescence intensity of individual cells was measured using LSM-FCS software.

### Measurements of mitochondrial membrane potential* Ψ*_m_

To estimate mitochondrial membrane potential* Ψ*_m_ in control and Zn-treated haemocytes, MitoProbe™ JC-1 Assay Kit was used (Molecular Probes, USA). JC-1 (5,59,6,69-tetrachloro-1,19,3,39-tetraethylbenzimidazolcarbocyanine iodide) is a potentiometric mitochondrial fluorochrome that aggregates in intact and negatively charged mitochondria emitting red fluorescence, whereas in cells with depolarized mitochondria, JC-1 is present in a form of cytoplasmic monomer showing green fluorescence. All experimental groups of haemocytes were pelleted at 1,500×*g* for 15 min at 22°C and suspended in 2 μM solution of JC-1 dissolved in HBSS. The cells were stained for 30 min in darkness, centrifuged, resuspended in HBSS, and transferred into 96-well plates. As the positive control, cells stained with JC-1 indicator were additionally treated with 0.5 mM of CCCP (HBSS solution) that disrupts mitochondrial membrane potential. The fluorescence intensity of JC-1 aggregates was measured with a fluorimeter (GloMax^®^-Multi Microplate Multimode Reader, Promega, USA). The collected data were normalized to the control (Zn-untreated) group of cells.

### Determination of cytoplasmic calcium level

A calcium indicator Oregon Green^®^ 488 BAPTA-1, AM (O6807, Molecular Probes, USA) was used to estimate changes in [Ca^2+^]_*i*_ in Zn-treated haemocytes and in the control ones. The cells were loaded with 10 μM solution of Oregon Green in HBSS for 30 min in darkness followed by propidium iodide staining (20 μg/ml, 2 min, BD Bioscience Pharmigen, USA) to distinguish between necrotic cells/cell debris and healthy cells. Haemocyte suspensions, 100 μl each, were transferred to cytometric tubes (BD Falcon™, USA), filled up to 600 μl with HBSS and the fluorescence of Oregon Green intensity was measured by means of a flow cytometer (FACSCalibur, BD Instruments, Franklin Lakes, NJ USA). The obtained data were analyzed using Cell Quest (BD, USA) and WinMDI 2.8 softwares (Gregori et al. 2004).

### Statistical analysis

Statistical analysis of data was performed using the Shapiro–Wilk W test for normality and the ANOVA or the non-parametric Kruskall–Wallis test (one-way test) followed by the Duncan post hoc test at *p* = 0.05. Additionally, outliers were detected and removed from the dataset on the basis of the Grubb’s test at *p* = 0.05. All data were plotted as mean ± SE.

## Results

### Intracellular Zn^2+^ concentration

**Fig. 1:**
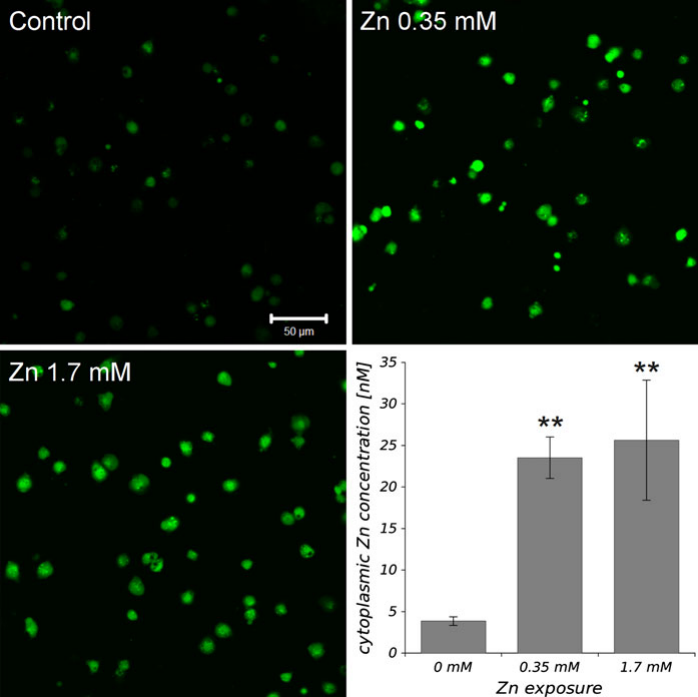
Representative confocal images obtained from haemocytes loaded with zinc-specific fluorochrome FluoZin-3 AM, not exposed to Zn ions (control) and treated for 3 h with 0.35, and 1.7 mM of Zn^2+^. The* histogram* represents the average values (±SE) of free Zn ion concentrations (nM) in the fruit fly’s haemocytes calculated on the basis of fluorescence intensity of FluoZin-3. **Statistically significant differences between experimental groups and control (*p* < 0.01, *N* = 3)

The analysis of FluoZin-3 fluorescence intensity in haemocytes, from the third instar larvae of *D. melanogaster* treated with Zn^2+^, revealed significantly higher concentration of Zn ions in the cytoplasm when compared with the control, the cells non-exposed to Zn^2+^ (Fig. 1). The average [Zn^2+^]_*i*_ in the control haemocytes was 3.85 nM, whereas in cells exposed to 0.35 and 1.7 mM of Zn^2+^ the concentration was 23.52 and 25.63 nM, respectively.

### Expression of *MtnA* and *MtnB*

**Fig. 2:**
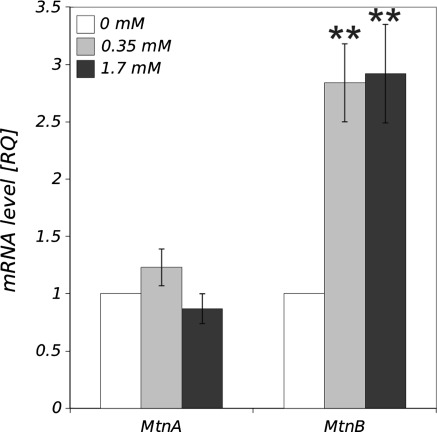
The effect of Zn treatment (0.35 and 1.7 mM) on *D. melanogaster MtnA* and *MtnB* gene expression in larval haemocytes quantified as mRNA levels using real-time PCR. Data are shown as RQ index calculated from ∆∆*C*_T_ method (mean ± SE) normalized to the control. **Statistically significant differences between experimental groups and control (*p* < 0.01, *N* = 6)

Real Time PCR quantification of *D. melanogaster* metallothionein A gene expression in larval haemocytes did not show any significant differences between control (untreated) and Zn-treated cells. On the other hand, both concentrations of Zn used in the study significantly enhanced metallothionein B gene expression. Relative quantity of the *MtnB* mRNA revealed that haemocytes from both experimental groups (0.35 and 1.7 mM) had nearly threefold higher *MtnB* expression than the control (Fig. 2).

### Activity of caspases and the level of DIAP1 protein

**Fig. 3:**
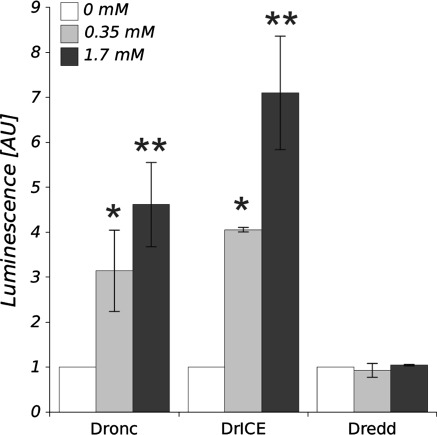
The activity of caspases DrICE, Dronc and Dredd in *D. melanogaster* haemocytes in the presence of 0.35 and 1.7 mM of Zn ions in comparison to the control. The results (means ± SE) are normalized to the level of caspase activity measured in the control cells. * and **Statistically significant differences between experimental groups and control for *p* < 0.05 and *p* < 0.01, respectively (*N* = 3)

**Fig. 4:**
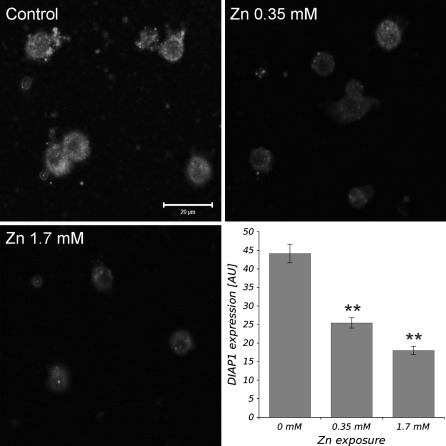
The results of DIAP1 expression measurements in *D. melanogaster* haemocytes treated for 3 h with 0.35 and 1.7 mM of zinc ions and in the control, Zn-untreated cells. Representative confocal images obtained from experimental and control groups of haemocytes. The intensity of red fluorescence exhibits the level of DIAP1 protein present in haemocyte cytoplasm. All micrographs were taken at the same imaging parameters using C-Apochromat 40×/1.2 W corr objective. The histogram represents mean values (±SE) of Cy-3 fluorescence bound to DIAP1 proteins after immunolabelling with anti-DIAP1 serum (see “Materials and methods”) in the fruit fly’s haemocytes. **Statistically significant differences between experimental groups and control (*p* < 0.01, *N* = 3)

The activities of caspases Dronc and DrICE in larval haemocytes of *D. melanogaster* were significantly higher in Zn-treated cells in comparison with the control (Fig. 3). The activity of Dronc increased threefold and over fourfold after treatment with 0.35 and 1.7 mM of Zn^2+^, respectively. The increase of activity of the effector caspase DrICE was dependent on Zn^2+^ concentration. We observed fourfold and sevenfold increase of DrICE activity in comparison with the control in media containing 0.35 and 1.7 mM of Zn^2+^, respectively. In contrast, any changes in the activity of Dredd were observed after applying both concentrations of Zn (Fig. 3). The examination of the caspase inhibitor DIAP1 in haemocytes showed that both zinc concentrations present in the cell environment significantly decrease the level of DIAP1. Two concentrations tested, 0.35 and 1.7 mM of Zn^2+^ reduced DIAP1 level by 58 and 41%, respectively when compared with the control (Fig. 4).

### Mitochondrial membrane potential* Ψ*_m_

**Fig. 5:**
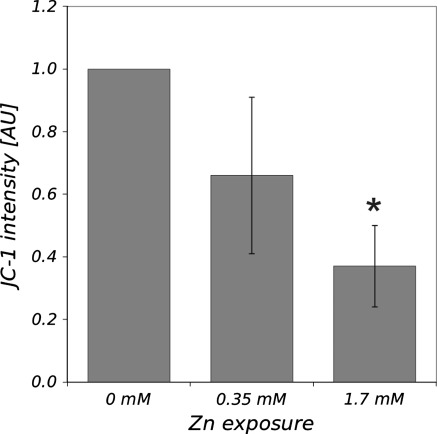
Changes in mitochondrial membrane potential in *D. melanogaster* haemocytes after their treatment with Zn ions. Haemocytes were stained with JC-1 indicator and intensity of red fluorescence was analyzed fluorometrically. Living haemocytes showed strong accumulation of JC-1 indicating intact mitochondria. After zinc treatment, disruption of mitochondrial membrane potential was observed leading to the significant decrease of JC-1 red fluorescence intensity. Data were normalized to the control samples and are shown as mean ± SE.* Asterisk* indicates statistically significant differences between experimental and control samples (*p* < 0.05, *N* = 3)

Monitoring changes in the mitochondrial membrane potential using JC-1 indicator, we found statistically significant decrease of *Ψ*_m_ only in cells exposed to 1.7 mM of Zn^2+^ (Fig. 5).

### Intracellular Ca^2+^ concentration

To evaluate the effect of Zn^2+^ on release of Ca^2+^ from cellular calcium stores, [Ca^2+^]_*i*_ concentration was measured with Oregon Green indicator by means of flow cytometry. However, no changes were observed in both Zn-treated groups of haemocytes in comparison with the control.

## Discussion

In the present study we found that Zn ions influence PCD in haemocytes obtained from the third instar larvae of *D. melanogaster*. We treated the cells with Zn^2+^ in concentrations of 0.35 and 1.7 mM, since in our previous study we observed that both concentrations efficiently affect PCD in haemocytes and do not have necrotic effects on those cells (Filipiak et al. 2010). The application of ZnCl_2_ to the cell media resulted in rapid increase of intracellular level of free Zn ions, visualized with a specific free-zinc indicator FluoZin-3. The average concentration of [Zn^2+^]_*i*_ in haemocyte cytoplasm increased about sixfold in both concentration used (Fig. 1). It indicates that Zn ions are easily transported to cell and increase the pool of labile Zn^2+^ in the cytoplasm. This labile pool of Zn^2+^ participates in many cellular processes and [Zn^2+^]_*i*_ is under control of regulatory mechanisms. However, a dramatic increase or decrease of labile Zn ions may lead to pathological processes or cell death (Truong-Tran et al. 2000).

In *D. melanogaster* dMTF-1, like its mammalian counterpart MTF-1, regulates transcription of metallothioneins responsible for binding and sequestering metal ions during heavy metal stress (Balamurugan et al. 2004). Five members of the metallothionein family have already been identified in the fruit fly cells; MtnA, MtnB, MtnC, MtnD and MtnE (Atanesyan et al. 2011). Two of them, MtnA and MtnB are related to the metal metabolism but unlike in mammals, they play a minor role in cell protection during zinc toxicity. They rather bind other metals, especially copper, since they are classified as copper-thioneins (Egli et al. 2006). Zhang et al. (2001) have observed that dMTF-1 does not induce metallothionein transcription in *D. melanogaster* cells at the concentration of zinc that stimulates its production in mammalian cells. However, at high levels of Zn^2+^ (2 mM) that is lethal to mammalian cells, dMTF-1 stimulates metallothionein expression in *D. melanogaster*. Our results showed that MtnB expression increases, whereas MtnA mRNA level does not change (Fig. 2). It confirms the observations of other authors that the expression of MtnA increases in response to copper and MtnB in the presence of high concentrations of zinc or cadmium intoxication (Yepiskoposyan et al. 2006).

Our study demonstrates for the first time that *D. melanogaster* haemocytes utilize the caspase cascade to eliminate cells intoxicated with high concentration of Zn ions. The activity of caspase Dronc, the fruit fly counterpart of caspase-9, and the major effector caspase DrICE were significantly increased in zinc-treated cells (Fig. 3). It seems that the presence of active form of Dronc caspase promotes DrICE to proteolytic activity. Similarly, mammalian thymocytes show zinc-induced PCD in result of apoptosome formation and caspase-9 activation, followed by procaspase-3 cleavage to active form of caspase-3 (Mann and Fraker 2005).

The obtained results also support the hypothesis that Dredd activity is not required for PCD in *D. melanogaster* cells (Fig. 3). The exposure of haemocytes to zinc ions did not activate Dredd proteins, thus receptor-mediated cell death in the fruit fly seems to play a minor role in response to zinc as it has been found in mammalian cells. Moreover, it is known that receptor-mediated PCD pathway does not involve Relish inhibition—the fruit fly’s member of NFκB family of proteins (Salvesen and Abrams 2004; Hay and Guo 2006). In mammalian cells, however, it has been reported that zinc-induced caspase activation might rely on the inhibition of NFκB translocation to the nucleus (Ho et al. 2004) and in consequence, down-regulation of antiapoptotic proteins e.g., caspase inhibitors or antiapoptotic proteins from Bcl-2 family (Chang et al. 2006).

It is well known that caspase activity is directly regulated by inhibitor of apoptosis proteins IAPs (Salvesen and Abrams 2004). Domains called baculoviral IAP repeats (BIR), present in all noted IAPs, contain zinc atom which is required for their antiapoptotic activity (Shi 2007). It seems that IAPs may be potential targets of many PCD-inducers, including the excess of Zn ions in cytoplasm. It has been demonstrated by Ku et al. (2010) in mammalian cells that the exposure to zinc reduces the expression of survivin, a member of IAP family. Our study confirms that high concentration of Zn decreases the level of DIAP1 (Fig. 4), the fruit fly IAP1 protein that may activate Dronc and DrICE—caspases to begin apoptotic pathway.

## Conclusions

Our results indicate for the first time that an excess of free zinc ions triggers PCD of haemocytes isolated from the third instar larvae of *D. melanogaster*. Zinc enters the cells and despite the stimulation of metallotionein B expression, increases significantly the pool of labile intracellular zinc ions. In consequence, metal ions decrease mitochondrial membrane potential and activate Dronc and DrICE caspases, associated with the intrinsic pathway of PCD. The enhanced activity of both caspases seems to be related to the low level of DIAP1 proteins observed in haemocytes exposed to high concentration of Zn^2+^. We observed that neither receptor-mediated PCD that utilized caspase Dredd nor calcium ions, do play the crucial role in PCD stimulation of the fruit fly immune cells exposed to high Zn concentrations. The mechanism of concentration-dependent PCD regulation by Zn ions is not fully understood yet and requires further studies.
